# Inter-eye correlation analysis of 24-h IOPs and glaucoma progression

**DOI:** 10.1007/s00417-022-05651-4

**Published:** 2022-05-02

**Authors:** Mohamad Dakroub, Raoul Verma-Fuehring, Vaia Agorastou, Julian Schön, Jost Hillenkamp, Frank Puppe, Nils A. Loewen

**Affiliations:** 1 Department of Ophthalmology, University of Würzburg, Josef-Schneider-Straße 11, 97080 Würzburg, Germany; 2 Institute for Artificial Intelligence and Knowledge Systems, Department of Informatics, University of Würzburg, Würzburg, Germany; 3 Artemis Eye Centers of Frankfurt, Hanauer Landstraße 147-149, 60314 Frankfurt, Germany

**Keywords:** Glaucoma progression, Nycthemeral intraocular pressure, Right-left comparison, Laterality

## Abstract

**Purpose:**

To determine whether 24-h IOP monitoring can be a predictor for glaucoma progression and to analyze the inter-eye relationship of IOP, perfusion, and progression parameters.

**Methods:**

We extracted data from manually drawn IOP curves with HIOP-Reader, a software suite we developed. The relationship between measured IOPs and mean ocular perfusion pressures (MOPP) to retinal nerve fiber layer (RNFL) thickness was analyzed. We determined the ROC curves for peak IOP (T_max_), average IOP(T_avg_), IOP variation (IOP_var_), and historical IOP cut-off levels to detect glaucoma progression (rate of RNFL loss). Bivariate analysis was also conducted to check for various inter-eye relationships.

**Results:**

Two hundred seventeen eyes were included. The average IOP was 14.8 ± 3.5 mmHg, with a 24-h variation of 5.2 ± 2.9 mmHg. A total of 52% of eyes with RNFL progression data showed disease progression. There was no significant difference in T_max_, T_avg_, and IOP_var_ between progressors and non-progressors (all *p* > 0.05). Except for T_avg_ and the temporal RNFL, there was no correlation between disease progression in any quadrant and T_max_, T_avg_, and IOP_var_. Twenty-four-hour and outpatient IOP variables had poor sensitivities and specificities in detecting disease progression. The correlation of inter-eye parameters was moderate; correlation with disease progression was weak.

**Conclusion:**

In line with our previous study, IOP data obtained during a single visit (outpatient or inpatient monitoring) make for a poor diagnostic tool, no matter the method deployed. Glaucoma progression and perfusion pressure in left and right eyes correlated weakly to moderately with each other.

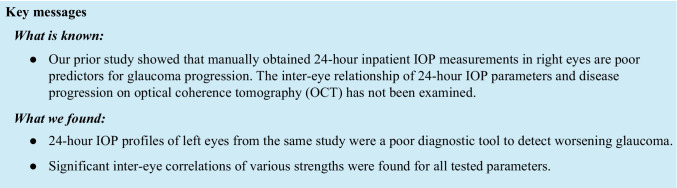

## Introduction

Open-angle glaucoma eventually becomes a bilateral disease in 50% of patients [1]. Several studies have investigated inter-eye relationships in glaucoma [2–7]. In patients with open-angle glaucoma in only one eye, chances of the contralateral eye developing this disease increase to about 40% after 2 years [7]. The probability of not just structural but functional loss in the form of a visual field defect is 25% at 5 years [2]. Visual field loss rates of both eyes are correlated in patients with bilateral glaucoma [3, 4] and progression in bilaterally affected eyes is faster than in patients with monocular disease [6]. Glaucomatous structure or functional deficits in one eye are predictive of contralateral eye retinal nerve fiber layer (RNFL) loss in the future [5]. One study showed that these seemingly healthy fellow eyes had an RNFL rate loss of 1.0 ± 0.2 µm per year, despite the absence of any clinical signs of glaucoma [5].

In several European countries, patients can be admitted for 24-h IOP monitoring to detect pressure peaks outside of regular office hours [8, 9] with the presumption that this is indicative of worsening or uncontrolled glaucoma. We recently created an extraction tool for manually charted IOP curves, HIOP-Reader [9, 10], and performed an exhaustive analysis of right eyes. We found that 24-h monitoring has limitations and, therefore, makes for a poor diagnostic tool in detecting glaucoma progression [9]. At the time, we had only included right eyes in our analysis of IOP and ocular perfusion parameters to reduce confounding variables.

The goal of this study was to examine the left eyes of the same dataset and to assess the inter-eye relationship of IOP, ocular perfusion pressure, and progression parameters. Our hypothesis was that while the use of 24-h IOP data may be flawed, these problems affect both eyes at the same time points, thereby still allowing an analysis of the inter-eye relationship that could be used to predict glaucoma. We expected IOP maxima, minima, perfusion pressures, and progression to be strongly correlated in right and left eyes.

## Methods

This retrospective study was carried out at the Department of Ophthalmology at the University of Würzburg. It adhered to the core principles stated in the Declaration of Helsinki. Informed consent was waived by the Institutional Review Board of the University of Würzburg due to its retrospective nature. Two hundred twenty-five charts of patients admitted for nycthemeral (24-h) IOP monitoring from 2017 to 2019 were reviewed. Diagnoses included primary open-angle glaucoma (POAG), pigmentary glaucoma (PG), juvenile glaucoma (JOAG), pseudoexfoliation glaucoma (PXG), and low-tension glaucoma (LTG). Patients with angle-closure, uveitic, neovascular glaucoma, and near-complete loss of the retinal nerve fiber layer on SDOCT were excluded.

Age, diagnoses, gender, slit lamp and fundoscopic examination findings, medications, central corneal thickness, surgical history, and family history were recorded. The historical 24-h IOP monitoring protocol in this hospital called for measurements at 10 AM, 2 PM, 5 PM, 9 PM, and 12 AM. All readings were acquired according to the protocol’s standard positions. The first four readings were measured in the sitting position using a Goldmann applanation tonometer (Haag-Streit, Köniz, Switzerland) while the fifth reading was acquired using a Perkins tonometer (Perkins MK3, Haag-Streit, Köniz, Switzerland) in the supine position. We wrote HIOP-Reader [9, 10], an image analysis program for high-speed extraction of data from manually drawn IOP charts. Analyzed IOP variables included T_avg_, T_min_, T_max_, and IOP_var_ (T_max_ − T_min_). Mean intraocular pressure (MOPP) was derived from the systolic (SBP) and the diastolic (DBP) blood pressures of patients recorded upon admission. It was calculated as follows:

$$\begin{array}{c}\mathrm{MOPP}=2/3\;(\mathrm{Mean}\;\mathrm{Arterial}\;\mathrm{Pressure}-\mathrm{IOP})\\\mathrm{where},\\\mathrm{Mean}\;\mathrm{Arterial}\;\mathrm{Pressure}=\mathrm{DBP}+1/3\;(\mathrm{SBP}-\mathrm{DBP})\end{array}$$

### Image analysis of manually recorded 24-h IOP profiles

All 24-h IOP profiles were recorded on A4 sheets equipped with graphs. Left and right eyes were plotted with different colors for differentiation. The evaluation was performed on standard consumer hardware from 2019 with a 2,4 GHz Quad-Core Intel Core i5-8279U CPU and 16 GB of random access memory. As described before [9], we used a Python-based program, *HIOP-Reader* [10], to extract examination date, patient name, and the IOP values on the y-axis with their corresponding time on the x-axis. In short, we used OpenCV [11] for image processing, Tesseract [12] for optical character recognition and TensorFlow [13], and scikit-learn [14] for machine learning. We also developed a graphical user interface for the program to allow for efficient editing and error correction. The image analysis was divided into three parts: preprocessing, value detection, and name and date extraction. The main goal of preprocessing was to detect the frame containing the IOP profile and crop the image to it.

To capture the date of the 24-h IOP profile, we applied a traditional machine learning approach. The numbers were predicted using a convolutional neural network trained on the Modified National Institute of Standards and Technology (MNIST) dataset. As the patient names were mostly recorded using machine-written labels, optical character recognition with Tesseract [15] could be used to extract all machine-written text on the form. To extract the IOP values entered into the profile, we detected the lines representing the different examination times using the Canny edge detection algorithm [16] and Hough line transformation [15]. We exploited the fact that all IOP values for the left eye were entered in red, while all values for the right eye were entered in blue and created color-specific masks. Since all images had the same format, the IOP value could be directly inferred from the pixel position of the detected entry. We then manually checked all entries for accuracy. A false entry was defined as an entry with an incorrect value; while a missed entry is an entry that was not detected by the software at all.

### Statistical analysis

#### Data management

Data were analyzed using the SPSS Statistics (Version 26, IBM, New York, USA). Percentages were calculated for categorical variables, while means and standard deviations were computed for continuous variables. The normality of data distribution was assessed using the Kolmogorov–Smirnov test. To examine the relationship between different IOP parameters, bivariate analyses were used and Spearman’s coefficients were reported. For relationships between dichotomous variables (progression versus no progression) and continuous variables, a binomial logistic regression was deployed. Linear regression was used for two continuous variables. Means were compared using the Wilcoxon signed-rank test. The correlation coefficients were interpreted using the generally considered classification of − 1 being the weakest and + 1 being the strongest correlation [17]. A *p*-value of 0.05 or less was considered statistically significant.

#### OCT and glaucoma progression analysis

Spectral Domain OCT equipped with the software “Glaucoma Module Premium Edition” (SPECTRALIS OCT, Heidelberg Engineering GmbH, Heidelberg, Germany) was used to assess disease progression by measuring the retinal nerve fiber layer (RNFL) thickness (in micrometers) of the peripapillary sectors. Progression was calculated both as a dichotomous and a continuous variable using a dedicated commercial software (HEYEX Version 2.4.1., Heidelberg Engineering GmbH, Heidelberg, Germany) by comparing the rate of RNFL loss to that of a normal age-related decline. The relationship between the slope of the RNFL loss and various continuous variables was assessed using linear regression.

## Results

### Demographics

Table 1 shows the demographics of patients included with their right and left eyes. In total, 217 left eyes were included in the statistical analysis. Females represented 60% of the cohort and were significantly older than males (age: 77.2 ± 9.7 years vs 72.7 ± 12.7 years, for females and males, respectively, *p*-value = 0.005). Glaucoma types included were: POAG (*n* = 130, 60%), PXG (*n* = 39, 18%), LTG (*n* = 41, 18.9%), PG (*n* = 4, 1.8%), and JOAG (*n* = 3, 1.4%). Thirty-eight patients (17.5%) were not taking any drops in their left eyes, while over a quarter (26.8%) were taking 4 medication drops at baseline. The most common types of medication were prostaglandins (75.6%), followed by carbonic anhydrase inhibitors (62.2%), alpha agonists (51.2%), and beta-blockers (44.7%). The average central corneal thickness (CCT) was 533.4 ± 37.7 µm, with no significant difference between genders (*p* = 0.9). The average MOPP was 59.5 ± 9.3 mmHg.

**Table 1:** Demographic parameters of all included patients

Age (years)	75.4 ± 11.2
Female	131 (60%)
Male	86 (40%)
# drops	R: 2.3 ± 1.4 L: 2.3 ± 1.4
# surgeries per eye	R: 0.6 ± 0.7 L: 0.6 ± 0.8
CCT (µm)	R: 535.5 ± 35.2 L: 533.4 ± 37.7
MOPP (mmHg)	R: 59.2 ± 9.0 L: 59.5 ± 9.3

#, number of; *R*, right eye; *L*, left eye; *CCT*, central corneal thickness; *MOPP*, mean ocular perfusion pressure

### IOP data extraction

**Fig. 1:**
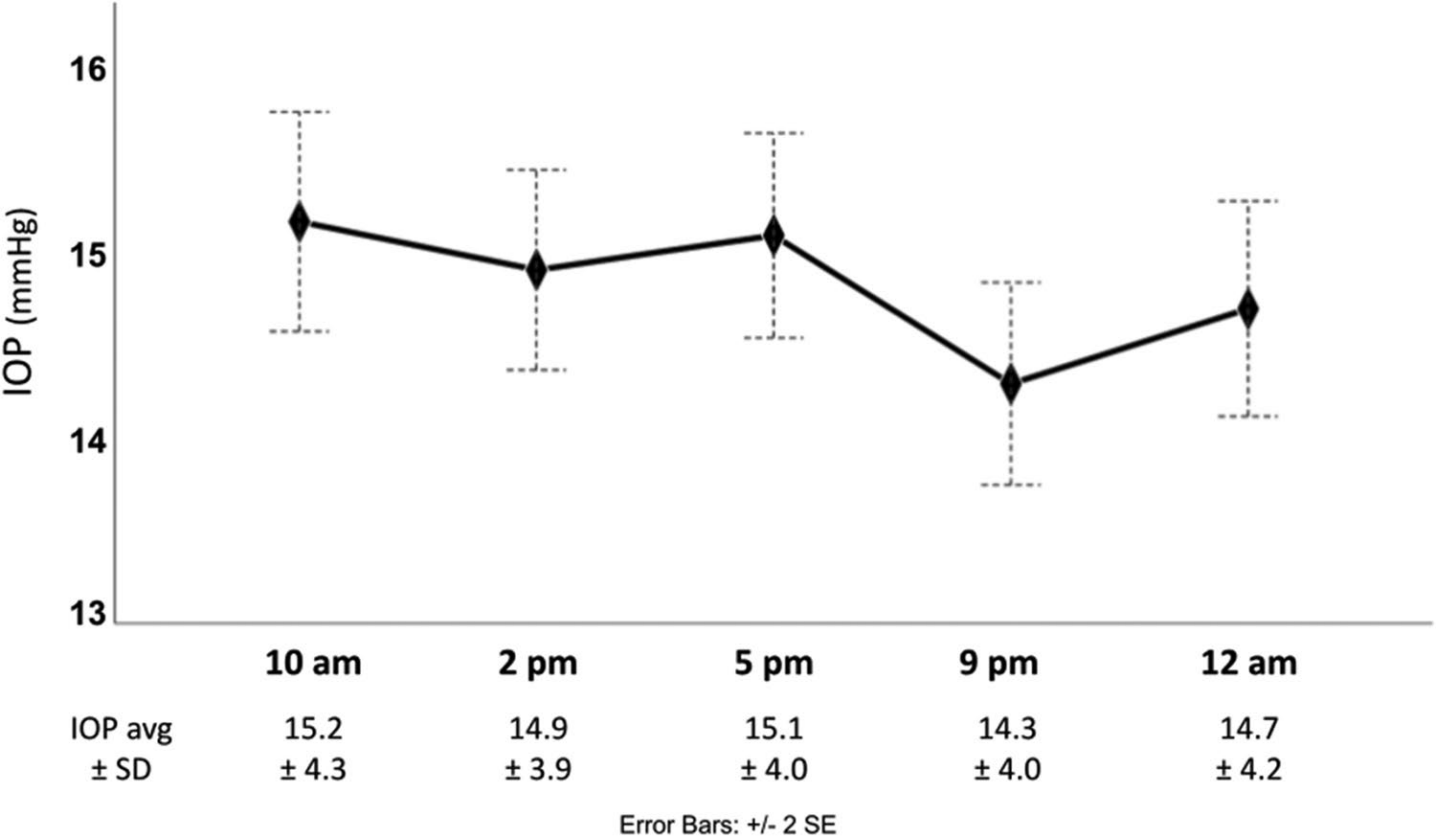
Average IOP values measured at the 5 nycthemeral monitoring protocol times. The peak IOP was recorded at 10 AM and the trough at 9 PM

Unlike right eyes that had their IOP values recorded in dark blue [9], left eye values were drawn into the chart using a red pencil. When using the HIOP-Reader on 100 IOP curves, an average of 8.4 entries per patient were recorded with a mean processing time of 3.6 ± 0.8 s per curve. The IOP data extraction was accurate, with an average of 0.5 falsely detected entries and 0.3 undetected entries per curve. The overall average IOP was 14.8 ± 3.5 mmHg. IOPs of right and left eyes (Table 2) were not statistically different from 5 PM through 12 AM but differed slightly for the 2 PM and 10 AM values (*p* = 0.03 for both) (Table 2). Figure 1 shows the average IOP values of the left eyes measured at the five 24-h monitoring protocol times. The means were comparable, with no significant differences between them (*p* = 0.18). T_max_, T_min_, and IOP_var_ were 17.4 ± 4.4 mmHg, 12.2 ± 3.1 mmHg, and 5.2 ± 2.9 mmHg, respectively. All 3 parameters were significantly correlated to MOPP (all *p*-values < 0.05).

**Table 2:** A comparison of IOP values between left and right eyes across different time-points

	Right (*n* = 225)	Left (*n* = 217)	*p*-value
IOP_10am_ (mmHg)	15.8 ± 5.1	15.2 ± 4.3	0.03*
IOP_2pm_ (mmHg)	15.5 ± 4.7	14.9 ± 3.9	0.03*
IOP_5pm_ (mmHg)	15.4 ± 4.6	15.1 ± 4.0	0.321
IOP_9pm_ (mmHg)	14.5 ± 4.7	14.3 ± 4.0	0.25
IOP_12am_ (mmHg)	14.8 ± 4.4	14.7 ± 4.2	0.33

### OCT Progression Data

RNFL Progression data from the Heyex software was available for 112 out of 217 eyes. These were divided into POAG (67, 59.8%), PXG (20, 17.9%), NTG (20, 17.9%), PG (2, 1.7%), and JOAG (3, 2.7%). Fifty-nine eyes (52.7%) showed a significant progression in at least one quadrant. Out of these, 33 (55.9%) had POAG, 14 (23.8%) and 10 (16.9%) had NTG and PXG, respectively. Only 1 (1.7%) patient progressed in each of PG and JOAG. More patients had a progression in the global peripapillary (32.1%) and the temporal superior (32.1%) sectors than in the temporal (23.2%) and temporal inferior (28.6%) sectors. Demographic parameters between progressors and non-progressors were comparable (Table 3). There were no significant differences in T_max_, T_avg_, and IOP_var_ between progressors and non-progressors (all *p*-values > 0.05). Except for T_avg_ and the temporal RNFL, there was no correlation between disease progression (rate of RNFL loss) in any quadrant and T_max_, T_avg_, and IOP_var_. Furthermore, a higher IOPvar was found to be associated with reduced disease progression in the temporal quadrant (odds ratio: 0.80, *p* = 0.04). MOPP was found to be correlated to disease progression only in the temporal sector (*p* = 0.04).

**Table 3:** Comparison of the demographic parameters of progressors and non-progressors in left eyes

Parameters	Progressors (*n* = 59)	Non-progressors (*n* = 53)	*p*-value
Age (years)	72.6 ± 11.1	73.7 ± 12.6	0.46
Female Male	37 (62.7%) 22 (37.3%)	27 (50.9%) 26 (49.1%)	0.25
# drops	2.3 ± 1.3	2.5 ± 1.5	0.34
# surgeries	0.7 ± 0.8	0.9 ± 0.9	0.49
T_avg_ (mmHg)	13.7 ± 2.5	14.4 ± 3.7	0.87
T_max_ (mmHg)	16.1 ± 3.1	16.9 ± 4.4	0.88
IOP_var_ (mmHg)	4.7 ± 2.3	5.0 ± 2.5	0.93
MOPP (mmHg)	61.0 ± 8.7	59.7 ± 9.8	0.92

#, number of; *T*_*avg*_, average IOP; *T*_*max*_, maximum IOP; *IOP*_*var*_, IOP variation; *MOPP*, mean ocular perfusion pressure

**Fig. 2:**
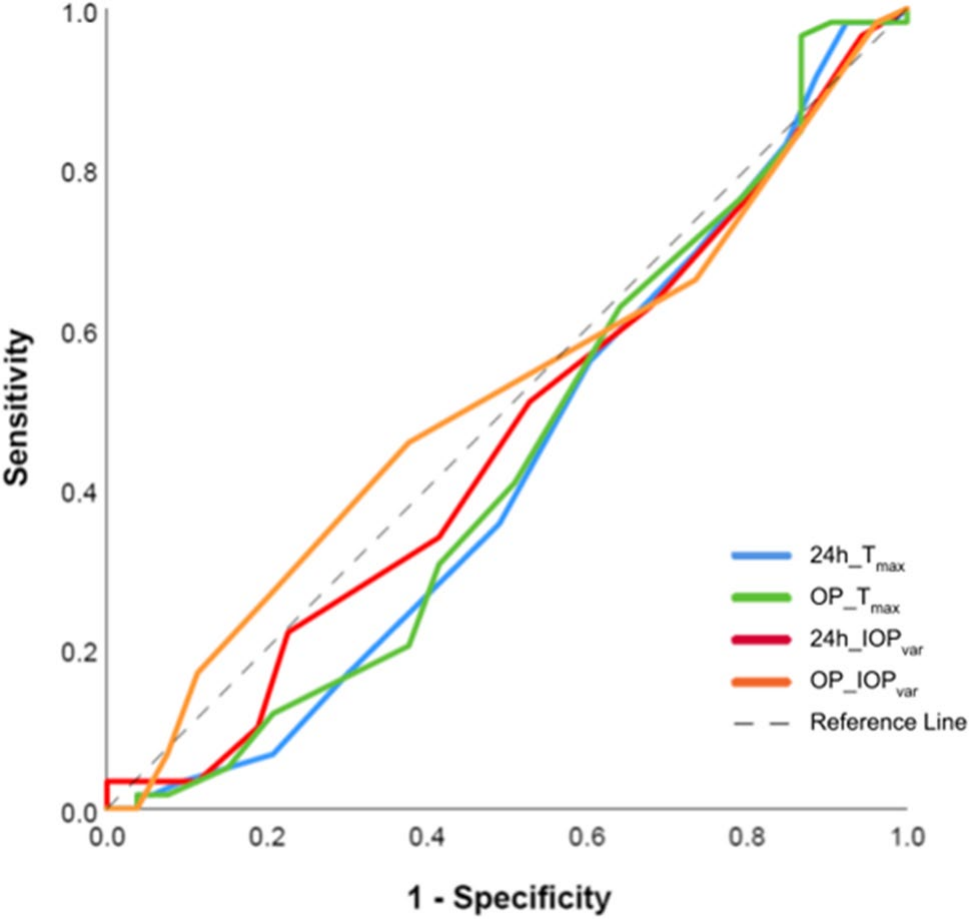
Receiver Operating Characteristic (ROC) curve for outpatient and 24-h parameters, T_max_ and IOP_var_, in detecting disease progression. As displayed, all plotted curves lie close to the reference line and expose the poor utility of these parameters for this purpose

Table 4 shows the sensitivity and specificity of using the historical cut-off points (T_max_) of 15 and 22 mmHg in detecting glaucoma progression for both 24-h and outpatient values (10 AM, 2 PM and 5 PM values only). These cut-off points revealed an unsatisfactory sensitivity–specificity combination in both groups. Figure 2 depicts the receiver operating characteristic (ROC) curve for the outpatient and 24-h parameters, T_max_ and IOP_var_, in detecting disease progression. All plotted curves lie in proximity to the reference line and demonstrate the poor utility of these parameters for this purpose.

**Table 4:** Utility of cut-off IOP_max_ values with sensitivity and specificity determined for cut-off points 15 and 22 mmHg for detecting glaucoma progression in left eyes

Cut-off value (IOP_max_)	Parameter	24-h IOP	OP-IOP	Difference
15 mmHg	Sensitivity Specificity	69.5% 26.4%	62.7% 35.8%	6.8% − 9.4%
22 mmHg	Sensitivity Specificity	3.4% 90.6%	1.7% 94.3%	1.7% − 3.7%

*OP-IOP*, taking into account only IOP measurements during outpatient hours (10 AM, 2 PM, 5 PM)

### Inter-eye analysis

T_max_ and IOP_var_ were both significantly greater in right eyes. T_max_ had a value of 19.3 ± 5.4 mmHg in right eyes and 17.4 ± 4.3 mmHg in left eyes (*p* < 0.001). For IOP_var_, these values were 6.9 ± 4.2 mmHg and 5.2 ± 2.9 mmHg, respectively (*p* < 0.001). There were no significant differences between left and right eyes in the other recorded parameters (T_avg_ and T_min_) and in the number of disease progressors. All right IOP parameters (T_max_, IOP_var_, T_avg_, and T_min_) showed moderate to strong positive correlations to their contralateral counterparts (Table 5). MOPP was very strongly correlated in both eyes; however, RNFL quadrants showed only a weak to moderate association (Table 5). Moreover, 74% of those who had a progression in the left eye and 67% of those who had a progression in the right eye also had a progression in the fellow eye. Using an adjusted chi-squared test that takes inter-ocular dependency into consideration, there was a significant moderate correlation between the progression of left and right eyes (*p* < 0.005, Phi = 0.32). Thirty-eight patients (35.2%) had the same worst quadrant bilaterally. Of those, TS was the worst quadrant in 20 (52.6%), TI in 15 (39.5%), and T in 3 (7.9%).

**Table 5:** Bivariate correlations of various measured parameters between left and right eyes. All correlations had a *p*-value < 0.05

Parameter	r-value	Strength of correlation
T_avg_	0.51	Moderate
IOP_var_	0.65	Strong
T_max_	0.78	Strong
T_min_	0.48	Moderate
# drops	0.76	Strong
# surgeries	0.48	Moderate
RNFLT loss		
G	0.43	Moderate
TS	0.33	Weak
T	0.29	Weak
TI	0.32	Weak
worst quadrant	0.36	Weak
MOPP	0.96	Very strong

*T*_*avg*_, average IOP; *IOP*_*var*_, IOP variation; *T*_*max*_, maximum IOP; *T*_*min*_, minimum IOP; #, number of; *RNFLT*, slope of retinal nerve fiber layer thickness decrease; *G*, global; *TS*, temporal-superior; *T*, temporal; *TI*, temporal-inferior

## Discussion

We recently examined the utility of 24-h (nycthemeral) IOP monitoring in detecting glaucoma progression in right eyes [9]. Here, we expanded on this research and analyzed this relationship in left eyes with a focus on inter-eye correlations. The HIOP-Reader will make for a useful tool to interrogate existing and newly acquired data in other eye clinics that routinely perform nycthemeral IOP measurements. Based on billing patterns of eye clinics in German-speaking countries [18, 19], we estimate that nycthemeral IOP curves must have been obtained about one million times over the last 100 years [8, 20–22]. Our study adds to the growing body of data suggesting that evidence supporting 24-h IOP profiles for identifying eyes at risk [8, 21–24] is surprisingly weak [25–27]. IOPs from left eyes were charted using a red pencil that has a lower contrast compared to the dark blue used for right eyes analyzed in our prior study, which makes it harder to delineate by automated image recognition [18]. Despite this, IOP data from left eyes was extracted equally well by our custom-made software, HIOP-Reader [9]. Goldmann measurements of left and right eyes had a statistically significant IOP difference of 0.6 mmHg at 10 AM and 2 PM. Although this difference was larger than the systematic error of 0.3 mmHg reported by Pekmezci et al. [19] for eyes measured first, we agree with these authors that the clinical significance of such a small difference is likely limited. Once again, we found no correlation between disease progression (rate of RNFL loss) and IOP parameters extracted from 24-h monitoring, except for one between T_avg_ and the temporal quadrant. This corroborates the conclusion that IOP variables, collected during a single visit should not be used to determine the disease trajectory.

The average patient age in our study was 75 years and 60% were women, similar to what other glaucoma studies have reported [19, 20]. Although means across at different times of the day did not differ significantly, we registered an IOP peak at 10 AM and a trough at 9 PM, similarly to our previous study. The 10 AM morning peak was delayed compared to peak IOPs reported in the literature [21–23]. However, our trough was in relatively good agreement with prior studies [21, 22, 24]. The overall average IOP value was within a low IOP range because most of our patients were receiving treatment.

Surprisingly, there were no statistical differences in the baseline characteristics of progressors and non-progressors and, except for T_avg_ in the temporal quadrant, no IOP parameters correlated to disease progression. The fact that IOP_var_ could not be linked to worsening glaucoma is contradictory to what has previously been reported on increased IOP fluctuations being associated with increased visual field defects in open-angle glaucoma (OAG) [25]. It is likely that too few readings and of variable quality were obtained by different on-call staff with various levels of experience. Nocturnal measurements were acquired using a Perkins handheld device, which is relatively user-dependent and necessitates experience. User-independent contact lens sensors which measure IOP fluctuations constantly over 24 h [25] or implantable sensors [26] are superior to our approach and allow IOP determination in a patient’s normal environment that also captures factors such as medication adherence.

Similarly, the use of a cut-off point exceeding the individual’s target IOP to predict progression did not appear to be any more useful. Our ROC curves for both left and right eyes show a poor utility of T_max_ for this purpose, which highlights the importance of combining multiple parameters to effectively manage glaucoma patients. We conclude that 24-h IOP measurements can somewhat guide therapy, but should not be used as a diagnostic tool to detect disease progression, even though IOP is a demonstrated cause of glaucoma and remains the only modifiable variable.

We found a positive correlation between IOP parameters in left and right eyes in the inter-eye correlation analysis. This is in agreement with the previously published literature that found IOP symmetry in both healthy and diseased fellow eyes [27–29]. A 2014 Chinese study on 397 healthy participants showed a very strong (*r* = 0.83) interocular correlation of IOP values [27]. In treatment-naive glaucoma patients, Sit et al. demonstrated a moderate association (*r* = 0.54) of diurnal IOP patterns between fellow eyes [28]. An even stronger inter-eye correlation was found by Mansouri et al. in a similar cohort using a contact lens sensor (*r* = 0.76) [29]. However, despite the potential existence of an interocular IOP association in glaucomatous eyes, it seems to be generally weaker than in healthy eyes [30, 31]. In fact, Williams et al. identified interocular IOP asymmetry as a significant risk factor for glaucoma. A premedication inter-eye asymmetry of > 6 mmHg in IOP values was associated with a 57% probability of having glaucoma [32].

In comparison to manually obtained IOP readings, OCT measurements are far less operator dependent. Our data revealed moderate but significant correlations for RNFL rate loss between right and left eyes in all quadrants. This finding is not surprising as several studies have previously described inter-eye correlations for disease progression [2–5, 33, 34]. For instance, a study by Chen et al. in 2002 revealed an interrelationship in visual field progression rates among fellow eyes with open-angle glaucoma [4]. Interestingly, a loss of fellow-eye RNFL thickness has even been demonstrated in glaucoma patients showing unilateral signs of disease such as optic disc changes and visual field defects [5].

Interocular MOPP was strongly correlated in our study (*r* = 0.96). This is unsurprising, as IOP_avg_ values did not differ significantly between both eyes, and values correlated with one another. Several studies have correlated glaucoma progression to a decrease in MOPP [35] but our study showed no significant association between the two. A reason for this may be the use of only one blood pressure measurement (on admission) and, thus, the inability to detect possible perfusion pressure fluctuations.

Our study had several limitations. Although the majority of glaucomas in our analysis were bilateral, some types, such as pseudoexfoliation glaucoma, are typically initially unilateral. We performed a subanalysis of PXG patients for this reason and found that the results were similar to the other patients. An important shortcoming of our study is the method of how the IOP was obtained and that patients may have had an improved medication adherence while under observation, commonly referred to as the observer or Hawthorne effect [36]. Another problem is the fact that IOP values and patterns of a single day are often not reproducible [37], an issue that can be overcome with implantable IOP sensors. The habitual, supine measurements were obtained with a Perkins tonometer, instead of a Goldmann tonometer that was used in the seated position during the day. Although both have been reported to be generally in good agreement [38], obese patients can have false high readings when seated in front of a Goldmann tonometer [39, 40]. Ideally, patients should have been measured with the same tonometry throughout the day, for instance with a pneumatonometer, commonly used in 24-h IOP sleep studies [41]. This practice has since been adopted in our hospital.

In conclusion, IOP parameters extracted from 24-h monitoring failed to predict disease progression in left eyes. IOPs and MOPPs of left and right eyes were positively correlated with each other and the vast majority of eyes, who had a significant RNFL loss in one eye, exhibited a similar pattern in the fellow eye.

## Data Availability

Data is available from the corresponding author on request.
